# Individual and Neighborhood Influences on the Relationship Between Waist Circumference and Coronary Heart Disease in the REasons for Geographic and Racial Differences in Stroke Study

**DOI:** 10.5888/pcd19.210195

**Published:** 2022-04-21

**Authors:** Anne H. Gaglioti, Desiree Rivers, Joanna Bryan Ringel, Suzanne Judd, Monika M. Safford

**Affiliations:** 1 National Center for Primary Care, Department of Family Medicine, Morehouse School of Medicine, Atlanta, Georgia; 2 Department of Community Health and Preventive Medicine, Morehouse School of Medicine, Atlanta, Georgia; 3 Department of Medicine, Division of General Internal Medicine, Weill Cornell School of Medicine, New York, New York; 4 Department of Biostatistics, School of Public Health, University of Alabama at Birmingham, Birmingham, Alabama

## Abstract

**Introduction:**

The objective of this study was to describe how the relationship between waist circumference and incident coronary heart disease (CHD) is influenced by individual and neighborhood factors in the REasons for Geographic and Racial Differences in Stroke (REGARDS) Study.

**Methods:**

REGARDS is a cohort study of 30,239 US adults. The primary exposure was sex-specific quartiles of waist circumference. Individual covariates included sociodemographic characteristics, health status, health behavior, and usual source of care. Neighborhood (ie, zip code–level) covariates included access to primary care, poverty, rurality, and racial segregation. The main outcome was incident CHD from baseline (2003) through 2017. We used descriptive statistics, Kaplan–Meier curves, and Cox proportional hazard models to analyze the overall sample and race–sex subgroups.

**Results:**

During the study period, 23,042 study participants had 1,499 CHD events. We found a higher risk of incident CHD in the upper quartile of waist circumference compared with the first quartile in all 4 race–sex subgroups except African American men, among whom we found no relationship between waist circumference and incident CHD. Covariates did not attenuate these relationships.

**Conclusion:**

In all groups except African American men, waist circumference in the highest quartile was associated with increased risk of incident CHD. Individual and neighborhood factors did not influence the relationship between waist circumference and development of CHD but differentially influenced incident CHD among race–sex subgroups.

**Summary**

**What is already known on this topic?**

Central adiposity and coronary heart disease (CHD) are influenced by individual and neighborhood characteristics, but it is unknown if the relationship between central obesity and development of CHD is influenced by these factors.

**What is added by this report?**

This study found that individual and neighborhood factors did not influence the relationship between baseline waist circumference and incident CHD but did differentially influence incident CHD in race–sex subgroups; we found no association between waist circumference and incident CHD among African American men.

**What are the implications for public health practice?**

Public health interventions for CHD prevention can be informed by these findings, which show that individual and neighborhood factors independently influence incident CHD differentially among race–sex subgroups.

## Introduction

Obesity is a well-established risk factor for coronary heart disease (CHD) (1–4) and has been associated with higher rates of death attributable to cardiovascular disease (CVD) (5). Additionally, there is consensus describing the association of waist circumference with cardiovascular events (6–8) and differences in the relationship between waist circumference and CVD mortality between non-Hispanic African American and non-Hispanic White populations (9). Waist circumference has been established as a better predictor of CHD risk than obesity, especially among older adults (10–12).

Although overall CHD mortality has declined in the US over the past 60 years (13), the US has persistent racial disparities in obesity rates (14) and CHD mortality (15,16). African Americans are 2 to 3 times more likely to be obese and have an average waist circumference that is substantially larger than the average waist circumference of the White population (14,17,18). At the individual level, both low socioeconomic status and African American race are associated with higher levels of stress, discrimination, elevated blood pressure, and mental disorders; worse access to and use of quality cardiovascular care; and poorer cardiovascular health outcomes (19,20). Although individual-level factors such as race, socioeconomic status, and mental health contribute to disparities in CHD, place-based social determinants of health, such as lack of access to healthy food options and quality health care and living in a poor or unsafe neighborhood, also independently contribute to the association between central adiposity and cardiovascular disparities (21–24). Socioeconomic disadvantage has been associated with poor health habits such as lack of physical activity, unhealthy eating, and smoking; increased depression; and higher levels of cardiovascular incidents (25). Even so, the relationship between waist circumference and CHD and neighborhood-level social determinants of health has not been as well studied.

This study builds on established complex relationships between individual and neighborhood risk profiles for CHD by asking the following research question: are relationships between waist circumference and CHD across subgroups influenced by both individual factors and neighborhood factors, just as CHD and obesity have been shown to be? Some neighborhood characteristics associated with obesity and CHD are access to primary care, poverty, rural status, racial segregation, and food access (26–30). Discerning how these characteristics influence the relationship between waist circumference and development of CHD while accounting for known individual-level factors associated with CHD could inform the effective design and implementation of neighborhood interventions to prevent CHD. Such interventions could potentially ameliorate upstream risks that likely contribute to the stark mortality gaps among racial groups in the US (31). We undertook this study to discern how neighborhood socioecologic factors influence the relationship between waist circumference and CHD in race–sex subgroups by using data from the longitudinal cohort in the REasons for Geographic and Racial Differences in Stroke (REGARDS) Study. We hypothesized that individual and neighborhood factors would differentially influence the relationship between waist circumference and CHD in race–sex subgroups and that African American participants would have less favorable neighborhood exposures than White participants.

## Methods

**Figure 1:**
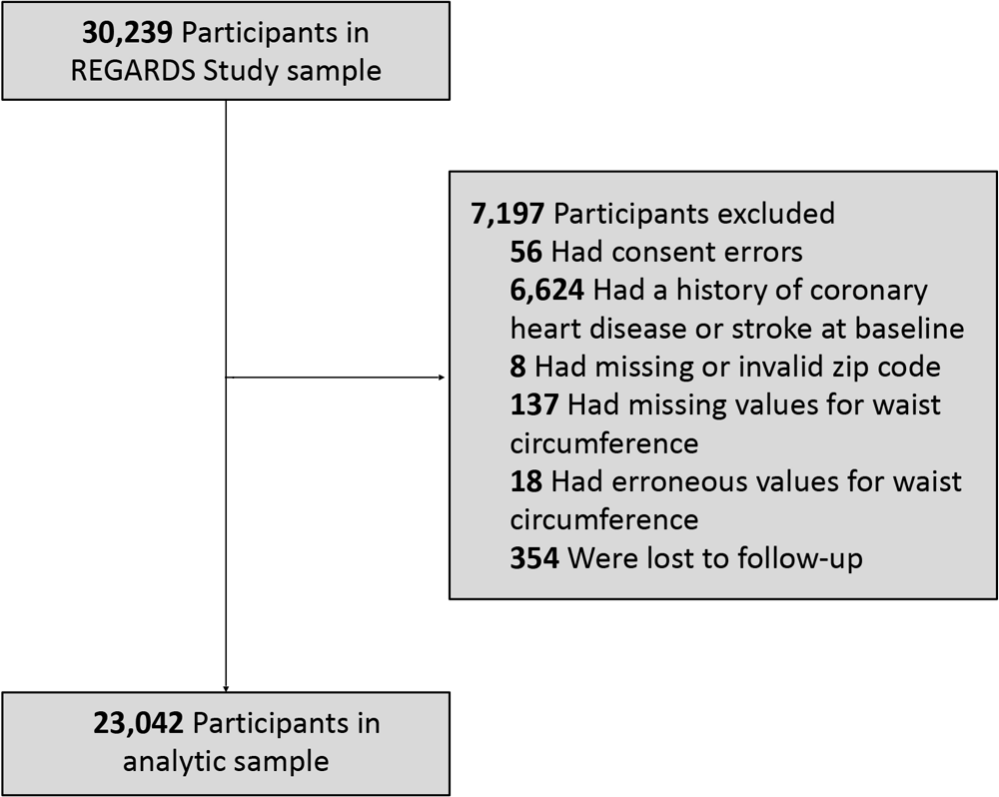
Exclusions in analytic sample for the REasons for Geographic and Racial Differences in Stroke (REGARDS) Study.

The design and methodology of the REGARDS study are described elsewhere (32). In brief, REGARDS is a population-based longitudinal cohort study of 30,239 non-Hispanic African American (hereinafter African American) and non-Hispanic White (hereinafter White) community-dwelling adults aged 45 years or older. Participants were recruited from the continental US from January 2003 through October 2007. The sampling scheme oversampled African Americans and residence in the southeastern US. The Stroke Buckle (coastal plain of Georgia, North Carolina, and South Carolina) was home to 20.9% of participants; 34.5% resided in the remainder of the Stroke Belt (remainder of Georgia, North Carolina, and South Carolina, and the southeastern states of Alabama, Arkansas, Louisiana, Mississippi, and Tennessee); and 44.5% lived in the other 42 continental states. Exclusion criteria were self-classified race other than African American or White, active cancer treatment, current or impending residence in a nursing facility due to limitation of long-term participation in the study, chronic illness that precluded long-term participation, or lack of English language proficiency. Data on baseline demographic characteristics and medical history were collected during a preliminary computer-assisted telephone interview followed by a home visit for a physical examination, consisting of anthropometric measurements, including waist circumference, medication inventory, electrocardiogram, phlebotomy, and urine collection. The study was approved by the institutional review boards at participating institutions, and written informed consent was obtained from participants. We excluded those with consent errors, a baseline history of stroke or CHD (based on self-report and evidence through electrocardiogram), missing or erroneous zip codes or waist circumference values, and participants lost to follow-up (Figure 1). The final analytic sample included 23,042 participants.

### Exposure

The exposure variable was baseline waist circumference; a tape measure was used to measure midway between the lowest rib and the iliac crest with the participant standing. Waist circumference was categorized in sex-specific quartiles. For men, the quartiles were defined by the following cut points: quartile 1, <36.0 inches; quartile 2, ≥36.0 to <38.5 inches; quartile 3, ≥38.5 to <42.0 inches; quartile 4, ≥42 inches. For women, the quartiles were defined by the following cut points: quartile 1, <32.0 inches; quartile 2, ≥32.0 to <36.0 inches; quartile 3, ≥36.0 to <40.0 inches; quartile 4, ≥40.0 inches. We undertook a sex-stratified analysis because definitions of normal waist circumference for men and women are different, and the cut points were also different between men and women.

### Outcome

The outcome variable was incident CHD. We included events through December 31, 2017. Living participants or their proxies were followed every 6 months by telephone, and medical records of reported hospitalizations were obtained. Medical records were reviewed by trained expert adjudicators who followed national guidelines to adjudicate myocardial infarction and CHD death (33). Myocardial infarction was adjudicated based on a clinical presentation suggestive of ischemia; a rising and/or falling pattern of cardiac biomarkers, most often troponin, with a peak at least twice the upper limit of normal; and electrocardiogram or imaging findings consistent with ischemia (34). We included definite or probable myocardial infarction events. Deaths were reported by next of kin or identified through the Social Security Death Index or National Death Index. Proxies were interviewed about the circumstances surrounding the death, including the presence of chest pain. Deaths were adjudicated with information obtained from multiple sources, including death certificates, autopsy reports, and medical records.

### Covariates

We selected individual covariates and ecologic characteristics according to established associations in the literature (Appendix).

**Individual characteristics.** Data for individual variables were obtained from the baseline data for REGARDS participants. Variables included race, sex, age, marital status, region of residence, educational attainment, annual household income, health insurance status, usual source of care, smoking status, diabetes diagnosis, hypertension diagnosis, and lipid disorder diagnosis. Marital status was categorized as married versus not married. Region of residence was defined as residence in the Stroke Belt, Stroke Buckle, or Non–Stroke Belt (32). Educational attainment was categorized as less than high school education, high school graduate, and college and above. Annual household income was categorized as earning >$35,000 or ≤$35,000 per year. Health insurance was dichotomized as having health insurance or not having health insurance. Having a usual source of care was defined as answering yes to the question “Is there a clinic or doctor who provides usual medical care for you?” Smoking status was defined as never, past, or current smoker. Diabetes diagnosis was defined as a fasting glucose of ≥126 mg/dL or nonfasting glucose of ≥200 mg/dL or self-reported use of medication to treat diabetes. Hypertension diagnosis was defined as systolic blood pressure ≥140 mm Hg or diastolic blood pressure ≥90 mm Hg or self-reported current medication use to control blood pressure. Dyslipidemia diagnosis was defined as total cholesterol ≥240 mg/dL, low-density lipoprotein cholesterol ≥160 mg/dL, high-density lipoprotein cholesterol ≤40 mg/dL, or self-reported use of medication to treat high cholesterol.

**Ecologic characteristics.** Ecologic covariates were assigned by using the participant’s baseline zip code of residence and included rural (vs urban) status, primary care physician (PCP) supply, zip code–level poverty, and area-level segregation. Rural status was assigned by the Rural Urban Commuting Area (RUCA) Code of the zip code, with RUCA codes 1.0, 1.1, 2.0, 2.1, 3.0, 4.1, 5.1, 7.1, 8.1, and 10.1 categorized as urban and the remaining codes categorized as rural (35). PCP supply was measured as the number of PCPs per 100,000 population in the Primary Care Service Area (PCSA) in 2010 where the participant resided at baseline; data were obtained from the Health Services and Research Administration data warehouse. PCSAs are units of geography that have been defined for the entire US based on patterns of primary care utilization (36). Data on zip code–level poverty were obtained from the 2010 US Census and defined as the percentage of the population living at or below 200% of the federal poverty level. Both PCPs per 100,000 population and zip code–level poverty were measured in quintiles. We calculated zip code–level of segregation by using an index of dissimilarity for African American and White populations in the zip code; this index indicates the proportion of the population that would have to move into the zip code to have uniform distribution of the population by race across the zip code (37).

### Statistical analysis

Descriptive statistics were compared for the full analytic sample and across sex-specific waist circumference quartiles. In addition, we compared characteristics across waist circumference quartiles within each race–sex subgroup. We used a χ^2^ test to determine significant differences. For continuous variables, we calculated means and used analysis of variance to analyze significance. We used Kaplan–Meier curves to investigate the proportion of incident CHD events in the sample over time, by waist circumference quartile.

We then analyzed the association between waist circumference quartiles and incident CHD for the total sample and stratified by race–sex group (White male, White female, African American male, and African American female). We used Cox regression to compute hazard ratios (HRs) and 95% CIs both crudely and adjusted for covariates, which were entered into the model in stages. Model 1 adjusted for all demographic characteristics except income, and model 2 adjusted for all demographics including income. Then, ecologic variables were added to the model one at a time to determine their impact on the association between waist circumference and incident CHD, independent of individual-level characteristics. Model 3 adjusted for demographic covariates and zip code–level poverty alone; model 4 adjusted for demographic characteristics and PCP supply in PCSA alone. Demographic characteristics as well as both zip code–level poverty and PCP supply in PCSA were adjusted for in model 5. Finally, model 6 adjusted for demographic characteristics, zip code–level poverty, PCP supply in PCSA, rural residence, and index of dissimilarity. We calculated both unadjusted and fully adjusted HRs for the association between each ecologic variable and incident CHD. The fully adjusted model included all demographic covariates. We explored interactions between neighborhood-level variables (proportion living in poverty, access to PCP, racial segregation, and rural status) and waist circumference on CHD in the total sample and in race–sex-stratified models. Because *P* values for interactions of (poverty × waist circumference) approached significance for White and African American women, we conducted poverty-stratified models (median split in the proportion of residents in the zip code living in poverty) for White and African American women.

Significance for analyses was defined as a 2-tailed *P* value of <.05, and 95% CIs were calculated for all estimates. Covariates with the highest percentage of missing data were usual source of care (12%) and income (7%). All other variables had <5% missing. We used multiple imputation with chained equations and 23 imputations to account for missing covariates in all modeling (38). Data management and statistical analysis was carried out in Stata/MP version 14 (StataCorp LLC) and SAS version 9.4 (SAS Institute, Inc).

## Results

During the study period, 1,499 CHD events occurred among 23,042 participants. The overall sample was 41.7% African American and 58.5% female, and the mean age was 64.0 years (Table 1). Participants in the highest quartile waist circumference group (quartile 4) were more likely than the overall sample to be African American women (35.8% vs 26.8%, *P* < .001), have income ≤$35,000 annually (45.7% vs 39.9%, *P* < .001), have less than a high school education (14.3% vs 11.0%), and live in a high-poverty neighborhood (24.0% vs 20.0%, *P* < .001). Quartile 4 also had more incident CHD events, a higher proportion of unmarried individuals, and a higher prevalence of chronic disease risk factors for CHD including diabetes, hypertension, and lipid disorders than did participants in other quartiles and the overall sample. We observed a higher proportion of African American participants than White participants (50.9% vs 49.1%) in waist circumference quartile 4. As waist circumference quartiles increased, we found significantly more participants in each quartile with less than a high school education and more participants living at or below 200% of the federal poverty level.

**Table 1:** Characteristics of Sample, by Quartiles of Waist Circumference, of 23,042 REGARDS Study Participants Without Coronary Heart Disease or Stroke at Baseline^a^

Characteristic	Total sample (no. of events)	Quartile of waist circumference b				*P* value^c^
		Quartile 1	Quartile 2	Quartile 3	Quartile 4	
No. in sample (no. of CHD events from baseline through December 31, 2017)	23,042 (1,499)	5,557 (278)	5,776 (328)	5,355 (346)	6,354 (547)	—
**Individual**						
Age, mean (SD), y	64.0 (9.3)	63.8 (9.8)	64.6 (9.5)	64.4 (9.1)	63.2 (8.7)	<.001
Race–sex group						
White male	6,122 (26.6)	1,383 (24.9)	1,561 (27.0)	1,514 (28.3)	1,664 (26.2)	<.001
White female	7,307 (31.7)	2,384 (42.9)	1,948 (33.7)	1,518 (28.3)	1,457 (22.9)	
African American male	3,432 (14.9)	932 (16.8)	829 (14.4)	711 (13.3)	960 (15.1)	
African American female	6,181 (26.8)	858 (15.4)	1,438 (24.9)	1,612 (30.1)	2,273 (35.8)	
Region of residence^d^						
Non–Stroke Belt	10,244 (44.5)	2,427 (43.7)	2,585 (44.8)	2,387 (44.6)	2,845 (44.8)	.47
Stroke Belt	7,968 (34.6)	1,919 (34.5)	1,965 (34.0)	1,863 (34.8)	2,221 (35.0)	
Stroke Buckle	4,830 (21.0)	1,211 (21.8)	1,226 (21.2)	1,105 (20.6)	1,288 (20.3)	
Not married (vs any other marital status)	9,469 (41.1)	2,167 (39.0)	2,269 (39.3)	2,222 (41.5)	2,811 (44.2)	<.001
Has a diabetes diagnosis (vs not)^e^	4,158 (18.7)	363 (6.8)	631 (11.3)	1,034 (19.9)	2,130 (34.6)	<.001
Has a hypertension diagnosis (vs not)^f^	12,681 (55.1)	2,077 (37.4)	2,942 (51.0)	3,133 (58.6)	4,529 (71.4)	<.001
Has a lipid disorder diagnosis (vs not)^g^	12,150 (54.8)	2,185 (41.3)	3,014 (54.0)	3,109 (60.0)	3,842 (62.8)	<.001
Smoking status						
Never	10,970 (47.8)	2,802 (50.6)	2,785 (48.4)	2,496 (46.8)	2,887 (45.6)	<.001
Past	8,776 (38.2)	1,776 (32.1)	2,181 (37.9)	2,136 (40.1)	2,683 (42.4)	
Current	3,206 (14.0)	957 (17.3)	794 (13.8)	700 (13.1)	755 (11.9)	
Educational attainment						
Less than high school education	2,524 (11.0)	433 (7.8)	569 (9.9)	611 (11.4)	911 (14.3)	<.001
High school graduate	12,052 (52.3)	2,710 (48.8)	3,033 (52.5)	2,842 (53.1)	3,467 (54.6)	
College graduate and above	8,454 (36.7)	2,410 (43.4)	2,172 (37.6)	1,898 (35.5)	1,974 (31.1)	
Annual household income, $						
≤35,000	9,184 (39.9)	1,937 (34.9)	2,213 (38.3)	2,131 (39.8)	2,903 (45.7)	<.001
>35,000	11,042 (47.9)	2,880 (51.8)	2,835 (49.1)	2,576 (48.1)	2,751 (43.3)	
Refused	2,816 (12.2)	740 (13.3)	728 (12.6)	648 (12.1)	700 (11.0)	
Has health insurance	21,370 (92.8)	5,184 (93.4)	5,408 (93.7)	4,972 (92.9)	5,806 (91.5)	<.001
Does not have a clinic or doctor who provides usual medical care	4,374 (20.5)	1,038 (20.0)	1,078 (20.0)	1,047 (21.2)	1,211 (20.7)	.41
**Neighborhood**						
Quintiles of primary care providers per 100,000 population in Primary Care Service Area						
<45	4,486 (19.5)	1,027 (18.5)	1,110 (19.2)	1,036 (19.4)	1,313 (20.7)	.03
45–62	4,789 (20.8)	1,152 (20.7)	1,250 (21.7)	1,075 (20.1)	1,312 (20.7)	
63–79	4,431 (19.2)	1,100 (19.8)	1,124 (19.5)	1,048 (19.6)	1,159 (18.2)	
80–107	4,683 (20.3)	1,115 (20.1)	1,129 (19.6)	1,143 (21.4)	1,296 (20.4)	
≥108	4,636 (20.1)	1,160 (20.9)	1,156 (20.0)	1,048 (19.6)	1,272 (20.0)	
Quintiles of percentage of population in zip code living below 200% of the federal poverty level						
<20.4	4,610 (20.0)	1,380 (24.8)	1,252 (21.7)	1,006 (18.8)	972 (15.3)	<.001
20.4–31.3	4,652 (20.2)	1,163 (20.9)	1,190 (20.6)	1,114 (20.8)	1,185 (18.7)	
31.4–39.5	4,521 (19.6)	1,028 (18.5)	1,130 (19.6)	1,080 (20.2)	1,283 (20.2)	
39.6–49.1	4,633 (20.1)	1,014 (18.3)	1,146 (19.9)	1,085 (20.3)	1,388 (21.9)	
≥49.2	4,604 (20.0)	969 (17.4)	1,048 (18.2)	1,064 (19.9)	1,523 (24.0)	
Zip code–level segregation, mean (SD)^h^	61.0 (13.3)	60.3 (13.0)	61.2 (13.4)	61.2 (13.4)	61.3 (13.2)	<.001
Urban vs rural residence^i^						
Urban	20,613 (89.6)	5,012 (90.3)	5,173 (89.8)	4,785 (89.5)	5,643 (88.9)	.07
Rural	2,393 (10.4)	536 (9.7)	589 (10.2)	562 (10.5)	706 (11.1)	

Abbreviation: —, does not apply; CHD, coronary heart disease; REGARDS, REasons for Geographic and Racial Differences in Stroke.

a Participants were recruited and baseline data were collected from January 2003 through October 2007. All values are number (percentage) unless otherwise indicated.

b Quartile 1: women, <32.0 in; men, <36.0 in. Quartile 2: women, ≥32.0 to <36.0 in; men, ≥36.0 to <38.5 in. Quartile 3: women, ≥36.0 to <40.0 in; men, ≥38.5 to <42.0 in. Quartile 4: women, ≥40.0 in; men, ≥42.0 in.

c χ^2^ test used to assess significance for categorical variables; analysis of variance or Kruskal–Wallis used for continuous variables.

d Categorized as Stroke Belt (Alabama, Arkansas, Louisiana, Mississippi, and Tennessee; and non–coastal plain of Georgia, North Carolina, and South Carolina); Stroke Buckle (coastal plain of Georgia, North Carolina, and South Carolina); non–Stroke Belt (all other states).

e Diabetes if fasting glucose ≥126 mg/dL, nonfasting glucose ≥200 mg/dL, or taking oral medication or insulin.

f Hypertension if systolic blood pressure ≥140 mm Hg, diastolic blood pressure ≥90 mm Hg, or self-reported current medication use to control blood pressure.

g Dyslipidemia defined as total cholesterol ≥240 mg/dL, low-density lipid cholesterol ≤40 mg/dL, or taking medication.

h An index of dissimilarity for African American and White populations in the zip code was used to indicate the proportion of the population that would have to move into the zip code to have uniform distribution of the population by race across the zip code (37).

i Rural–Urban Commuting Areas (RUCA) categorization (35): RUCA 1.0,1.1, 2.0, 2.1, 3.0, 4.1, 5.1, 7.1, 8.1, and 10.1 categorized as urban; RUCA 4.0, 4.2, 5.0, 5.2, 6.0, 6.1, 7.0, 7.2, 7.3, 7.4, 8.0, 8.2, 8.3, 8.4, 9.0, 9.1, 9.2, 10.0, 10.2, 10.3, 10.4, 10.5, and 10.6 categorized as rural.

Among race–sex subgroups, we found significant differences in self-report of a usual source of health care: 30.6% of African American men reported no usual source of care, compared with 20.0% of African American women, 21.9% of White men, and 15.2% of White women (*P* < .001) (Table 2). A greater percentage of African American men and women lived in areas with the most PCP supply, but they were more likely to report not having a usual source of health care. We also observed differences in the rural/urban status of racial groups: 94% of African American participants lived in urban areas, whereas 86% of White participants lived in urban areas.

**Table 2:** Characteristics of Sample, by Race–Sex Group, of 23,042 REGARDS Study Participants Without Coronary Heart Disease or Stroke at Baseline^a^

Characteristic	White male	White female	African American male	African American female	*P* value^b^
No. in sample (no. of CHD events from baseline through December 31, 2017)	6,122 (535)	7,307 (357)	3,432 (281)	6,181 (326)	<.001
**Individual**					
Age, mean (SD), y	64.7 (9.1)	64.0 (9.5)	63.6 (9.2)	63.4 (9.3)	<.001
Race–sex group					
White male	6,122 (100.0)	0	0	0	<.001
White female	0	7,307 (100.0)	0	0	
African American male	0	0	3,432 (100.0)	0	
African American female	0	0	0	6,181 (100.0)	
Region of residence^c^					
Non–Stroke Belt	2,804 (45.8)	2,807 (38.4)	1,730 (50.4)	2,903 (47.0)	<.001
Stroke Belt	2,107 (34.4)	2,625 (35.9)	1,151 (33.5)	2,085 (33.7)	
Stroke Buckle	1,211 (19.8)	1,875 (25.7)	551 (16.1)	1,193 (19.3)	
Not married (vs any other marital status)	1,002 (16.4)	3,215 (44.0)	1,147 (33.4)	4,105 (66.4)	<.001
Marital status					
Married	5,120 (83.6)	4,092 (56.0)	2,285 (66.6)	2,076 (33.6)	<.001
Divorced	407 (6.6)	1,163 (15.9)	481 (14.0)	1,475 (23.9)	
Widowed	333 (5.4)	1,670 (22.9)	318 (9.3)	1,822 (29.5)	
Single	222 (3.6)	306 (4.2)	214 (6.2)	541 (8.8)	
Other	40 (0.7)	76 (1.0)	134 (3.9)	267 (4.3)	
Has a diabetes diagnosis (vs not)^d^	837 (14.0)	791 (11.2)	922 (27.9)	1,608 (27.3)	<.001
Has a hypertension diagnosis (vs not)^e^	2,826 (46.2)	3,311 (45.4)	2,221 (64.8)	4,323 (70.0)	<.001
Has a lipid disorder diagnosis (vs not)^f^	3,906 (65.4)	3,524 (50.1)	1,871 (56.9)	2,849 (48.6)	<.001
Smoking status					
Never	2,408 (39.5)	4,004 (55.0)	1,225 (35.9)	3,333 (54.2)	<.001
Past	2,997 (49.1)	2,357 (32.4)	1,537 (45.0)	1,885 (30.6)	
Current	694 (11.4)	923 (12.7)	652 (19.1)	937 (15.2)	
Educational attainment					
Less than high school education	344 (5.6)	463 (6.3)	594 (17.3)	1,123 (18.2)	<.001
High school graduate	2,663 (43.5)	4,106 (56.2)	1,873 (54.6)	3,410 (55.2)	
College graduate and above	3,114 (50.9)	2,733 (37.4)	963 (28.1)	1,644 (26.6)	
Annual income, $					
≤35,000	1,545 (25.2)	2,735 (37.4)	1,505 (43.9)	3,399 (55.0)	<.001
>35,000	4,044 (66.1)	3,460 (47.4)	1,586 (46.2)	1,952 (31.6)	
Refused	533 (8.7)	1,112 (15.2)	341 (9.9)	830 (13.4)	
Has health insurance	5,855 (95.7)	6,924 (94.8)	3,084 (90.0)	5,507 (89.2)	<.001
Does not have a clinic or doctor who provides usual medical care	1,276 (21.9)	1,053 (15.2)	924 (30.6)	1,121 (20.0)	<.001
**Neighborhood**					
Quintiles of primary care provider per 100,000 population in Primary Care Service Area					
<45	1,239 (20.2)	1,533 (21.0)	582 (17.0)	1,132 (18.3)	<.001
45–62	1,434 (23.4)	1,723 (23.6)	587 (17.1)	1,045 (16.9)	
63–79	1,240 (20.3)	1,541 (21.1)	591 (17.3)	1,059 (17.2)	
80–107	1,197 (19.6)	1,319 (18.1)	788 (23.0)	1,379 (22.3)	
≥108	1,012 (16.5)	1,190 (16.3)	878 (25.6)	1,556 (25.2)	
Quintiles of percentage of population in zip code living below 200% of federal poverty level					
<20.4	1,910 (31.2)	2,069 (28.3)	272 (7.9)	359 (5.8)	<.001
20.4–31.3	1,611 (26.3)	1,910 (26.2)	427 (12.5)	704 (11.4)	
31.4–39.5	1,126 (18.4)	1,463 (20.0)	705 (20.6)	1,227 (19.9)	
39.6–49.1	934 (15.3)	1,227 (16.8)	853 (24.9)	1,619 (26.2)	
≥49.2	539 (8.8)	635 (8.7)	1,168 (34.1)	2,262 (36.7)	
Zip code–level segregation, mean (SD)^g^	60.5 (13.6)	60.7 (13.6)	60.9 (12.8)	61.9 (12.7)	<.001
Urban (vs rural) residence of participant^h^	5,323 (87.0)	6,297 (86.3)	3,220 (94.1)	5,773 (93.6)	<.001

Abbreviation: REGARDS, REasons for Geographic and Racial Differences in Stroke.

a Participants were recruited and baseline data were collected from January 2003 through October 2007. All values are number (percentage) unless otherwise indicated.

b χ^2^ test used to assess significance for categorical variables; analysis of variance or Kruskal–Wallis used for continuous variables.

c Categorized as Stroke Belt (Alabama, Arkansas, Louisiana, Mississippi, and Tennessee; and non–coastal plain of Georgia, North Carolina, and South Carolina); Stroke Buckle (coastal plain of Georgia, North Carolina, and South Carolina); non–Stroke Belt (all other states).

d Diabetes if fasting glucose ≥126 mg/dL, nonfasting glucose ≥200 mg/dL, or taking oral medication or insulin.

e Hypertension if systolic blood pressure ≥140 mm Hg, diastolic blood pressure ≥90 mm Hg, or self-reported current medication use to control blood pressure.

f Dyslipidemia defined as total cholesterol ≥240 mg/dL, low-density lipid cholesterol ≤40 mg/dL, or taking medication.

g An index of dissimilarity for African American and White populations in the zip code was used to indicate the proportion of the population that would have to move into the zip code to have uniform distribution of the population by race across the zip code (37).

h Rural–Urban Commuting Areas (RUCA) categorization (35): RUCA 1.0,1.1, 2.0, 2.1, 3.0, 4.1, 5.1, 7.1, 8.1, and 10.1 categorized as urban; RUCA 4.0, 4.2, 5.0, 5.2, 6.0, 6.1, 7.0, 7.2, 7.3, 7.4, 8.0, 8.2, 8.3, 8.4, 9.0, 9.1, 9.2, 10.0, 10.2, 10.3, 10.4, 10.5, and 10.6 categorized as rural.

In unadjusted and full models for the entire sample and for White men, White women, and African American women, we found an increased risk of incident CHD in the highest quartile of waist circumference compared with the first quartile (Table 3). For the overall population, the unadjusted HR was 1.81 (95% CI, 1.56–2.09) and the fully adjusted HR was 1.44 (95% CI, 1.23–1.68) in the highest versus the lowest quartile for waist circumference. Both the crude and fully adjusted HRs were significant for White men (adjusted HR, 1.56; 95% CI, 1.20–2.04), White women (adjusted HR, 1.56; 95% CI, 1.14–2.12), and African American women (adjusted HR, 1.88; 95% CI, 1.25–2.83). We found no significant relationship between waist circumference and incident CHD in either unadjusted or adjusted analyses among African American men (adjusted HR, 0.90; 95% CI, 0.64–1.26). Neither individual sociodemographic variables, including CHD risk factors, nor ecologic covariates attenuated the relationship between waist circumference and incident CHD in the overall sample or in the race–sex stratified models.

**Table 3:** Hazard Ratios for Association of Waist Circumference Quartiles^a^ With Coronary Heart Disease, by Stratified Race–Sex Subgroups, Among 23,042 REGARDS Participants^b^

Model/waist circumference quartile	Total population, HR (95% CI)	White male, HR (95% CI)	White female, HR (95% CI)	African American male, HR (95% CI)	African American female, HR (95% CI)
No. in sample (no. of CHD events from baseline through December 31, 2017)	23,042 (1,499)	6,122 (535)	7,307 (357)	3,432 (281)	6,181 (326)
**Unadjusted model**					
Quartile 1	1 [Reference]	1 [Reference]	1 [Reference]	1 [Reference]	1 [Reference]
Quartile 2	1.13 (0.96–1.32)	1.20 (0.91–1.57)	1.14 (0.84–1.54)	1.01 (0.72–1.41)	1.16 (0.74–1.81)
Quartile 3	1.30 (1.11–1.52)	1.37 (1.05–1.78)	1.45 (1.07–1.97)	1.03 (0.73–1.45)	1.34 (0.87–2.06)
Quartile 4	1.81 (1.56–2.09)	1.78 (1.39–2.29)	2.21 (1.67–2.92)	1.10 (0.80–1.52)	2.27 (1.53–3.37)
**Model 1: Demographic characteristics^c^ except annual household income**					
Quartile 1	1 [Reference]	1 [Reference]	1 [Reference]	1 [Reference]	1 [Reference]
Quartile 2	1.02 (0.87–1.20)	1.15 (0.88–1.51)	0.94 (0.69–1.27)	0.92 (0.65–1.30)	1.06 (0.68–1.66)
Quartile 3	1.10 (0.93–1.29)	1.23 (0.93–1.61)	1.09 (0.80–1.49)	0.93 (0.65–1.33)	1.14 (0.74–1.76)
Quartile 4	1.45 (1.24–1.70)	1.57 (1.20–2.05)	1.56 (1.14–2.12)	0.89 (0.64–1.26)	1.87 (1.24–2.81)
**Model 2: Demographic characteristics^c^ including annual household income**					
Quartile 1	1 [Reference]	1 [Reference]	1 [Reference]	1 [Reference]	1 [Reference]
Quartile 2	1.02 (0.87–1.20)	1.15 (0.88–1.51)	0.94 (0.69–1.27)	0.93 (0.66–1.31)	1.05 (0.67–1.65)
Quartile 3	1.10 (0.93–1.29)	1.23 (0.94–1.61)	1.10 (0.80–1.50)	0.95 (0.66–1.36)	1.13 (0.73–1.75)
Quartile 4	1.45 (1.24–1.69)	1.57 (1.20–2.05)	1.57 (1.15–2.14)	0.91 (0.64–1.27)	1.85 (1.23–2.78)
**Model 3: Demographic characteristics^c^ + zip code tabulation area income^d^**					
Quartile 1	1 [Reference]	1 [Reference]	1 [Reference]	1 [Reference]	1 [Reference]
Quartile 2	1.02 (0.87–1.20)	1.15 (0.88–1.51)	0.94 (0.69–1.27)	0.92 (0.66–1.30)	1.06 (0.67–1.65)
Quartile 3	1.09 (0.93–1.28)	1.22 (0.93–1.61)	1.09 (0.80–1.49)	0.95 (0.66–1.36)	1.13 (0.73–1.74)
Quartile 4	1.44 (1.23–1.68)	1.56 (1.19–2.03)	1.56 (1.14–2.12)	0.90 (0.64–1.27)	1.85 (1.23–2.78)
**Model 4: Demographic characteristics^c^ + primary care provider supply in Primary Care Service Area**					
Quartile 1	1 [Reference]	1 [Reference]	1 [Reference]	1 [Reference]	1 [Reference]
Quartile 2	1.02 (0.87–1.20)	1.15 (0.88–1.52)	0.94 (0.69–1.27)	0.93 (0.66–1.31)	1.08 (0.69–1.69)
Quartile 3	1.10 (0.93–1.29)	1.23 (0.94–1.61)	1.10 (0.80–1.50)	0.94 (0.66–1.35)	1.16 (0.75–1.80)
Quartile 4	1.45 (1.24–1.69)	1.57 (1.20–2.06)	1.56 (1.14–2.12)	0.90 (0.64–1.27)	1.90 (1.26–2.85)
**Model 5: Demographic characteristics^c^ + zip code tabulation area^d^ + primary care provider supply in Primary Care Service Area**					
Quartile 1	1 [Reference]	1 [Reference]	1 [Reference]	1 [Reference]	1 [Reference]
Quartile 2	1.02 (0.87–1.20)	1.15 (0.88–1.52)	0.94 (0.69–1.27)	0.92 (0.65–1.30)	1.08 (0.69–1.69)
Quartile 3	1.09 (0.93–1.28)	1.22 (0.93–1.61)	1.09 (0.80–1.49)	0.94 (0.66–1.35)	1.16 (0.75–1.80)
Quartile 4	1.44 (1.23–1.69)	1.56 (1.20–2.04)	1.55 (1.14–2.12)	0.90 (0.64–1.26)	1.90 (1.26–2.86)
**Model 6: All individual and neighborhood variables^e^**					
Quartile 1	1 [Reference]	1 [Reference]	1 [Reference]	1 [Reference]	1 [Reference]
Quartile 2	1.02 (0.87–1.20)	1.16 (0.88–1.52)	0.94 (0.70–1.28)	0.92 (0.65–1.29)	1.07 (0.68–1.68)
Quartile 3	1.09 (0.93–1.28)	1.22 (0.93–1.60)	1.10 (0.80–1.50)	0.94 (0.66–1.35)	1.16 (0.75–1.79)
Quartile 4	1.44 (1.23–1.68)	1.56 (1.20–2.04)	1.56 (1.14–2.12)	0.90 (0.64–1.26)	1.88 (1.25–2.83)

Abbreviations: HR, hazard ratio; REGARDS, REasons for Geographic and Racial Differences in Stroke.

a Quartile 1: women, <32.0 in; men, <36.0 in. Quartile 2: women, ≥32.0 to <36.0 in; men, ≥36.0 to <38.5 in. Quartile 3: women, ≥36.0 to <40.0 in; men, ≥38.5 to <42.0 in. Quartile 4: women, ≥40.0 in; men, ≥42.0 in.

b Participants were recruited and baseline data were collected from January 2003 through October 2007.

c Demographics include race, sex, region of residence, age, marital status, diabetes diagnosis, hypertension diagnosis, lipid disorder diagnosis, smoking status, educational attainment, health insurance status, and usual source of care.

d Zip code tabulation area income defined as percentage of population living below 200% of federal poverty level in zip code.

e Ecologic variables include zip code tabulation area income, primary care provider supply in Primary Care Service Area, rural residence, and index of dissimilarity. An index of dissimilarity for African American and White populations in the zip code was used to indicate the proportion of the population that would have to move into the zip code to have uniform distribution of the population by race across the zip code (37).

For White women in quartile 4 for waist circumference living in areas where >35.8% of the population lives in poverty, the adjusted HR was 1.77 (95% CI, 1.07–2.94) versus White women in quartile 4 for waist circumference living in areas where ≤35.8% of the population lives in poverty (adjusted HR, 1.49; 95% CI, 1.00–2.21 (Table 4). For African American women in quartile 4 for waist circumference living in areas where >35.8% of the population lives in poverty, fully adjusted HRs were significant (adjusted HR, 2.10; 95% CI 1.29–3.24), while African American women in quartile 4 for waist circumference living in areas where ≤35.8% of the population lives in poverty did not have a significant adjusted HR for incident CHD.

**Table 4:** Fully Adjusted Hazard Ratios for the Association Between Waist Circumference and Coronary Heart Disease, Stratified by Zip Code–Level Poverty, Among White Women and African American Women Participating in the REGARDS Study

Quartile of waist circumference^b^	≤35.8% of Population in zip code lives in poverty^a^			>35.8% of Population in zip code lives in poverty		
	No. in sample (no. of CHD events)^c^	HR (95% CI)	*P* value	No. in sample (no. of CHD events)^c^	HR (95% CI)	*P* value
**White women**						
Quartile 1	1,624 (59)	1 [Reference]		760 (29)	1 [Reference]	
Quartile 2	1,299 (56)	1.06 (0.73–1.54)	.75	647 (25)	0.79 (0.46–1.36)	.40
Quartile 3	963 (41)	0.97 (0.64–1.46)	.87	555 (37)	1.33(0.80–2.19)	.27
Quartile 4	905 (59)	1.49 (1.00–2.21)	.05	551 (51)	1.77 (1.07–2.94)	.03
**African American women^d^**						
Quartile 1	214 (9)	1 [Reference]		642 (20)	1 [Reference]	
Quartile 2	415 (11)	0.54 (0.22–1.31)	.17	1,019 (45)	1.35 (0.79–2.29)	.27
Quartile 3	415 (16)	0.79 (0.34–1.82)	.58	1,195 (57)	1.34 (0.80–2.25)	.27
Quartile 4	528 (37)	1.52 (0.70–3.30)	.29	1,743 (130)	2.10 (1.29–3.24)	<.001

Abbreviations: CHD, coronary heart disease; HR, hazard ratio; REGARDS, REasons for Geographic and Racial Differences in Stroke.

a On the basis of data distribution, poverty level was a median split at >35.8% of the population in zip code living in poverty and ≤35.8% of the population in zip code living in poverty.

b Quartile 1: women, <32.0 in; Quartile 2: women, ≥32.0 to <36.0 in; Quartile 3: women, ≥36 to <40.0 in. Quartile 4: women ≥40 in.

c Participants were recruited and baseline data were collected from January 2003 through October 2007. Data on number of CHD events were collected through December 31, 2017.

d HRs for African American women should be interpreted with caution because of the small number of CHD events in each quartile.

**Figure 2:**
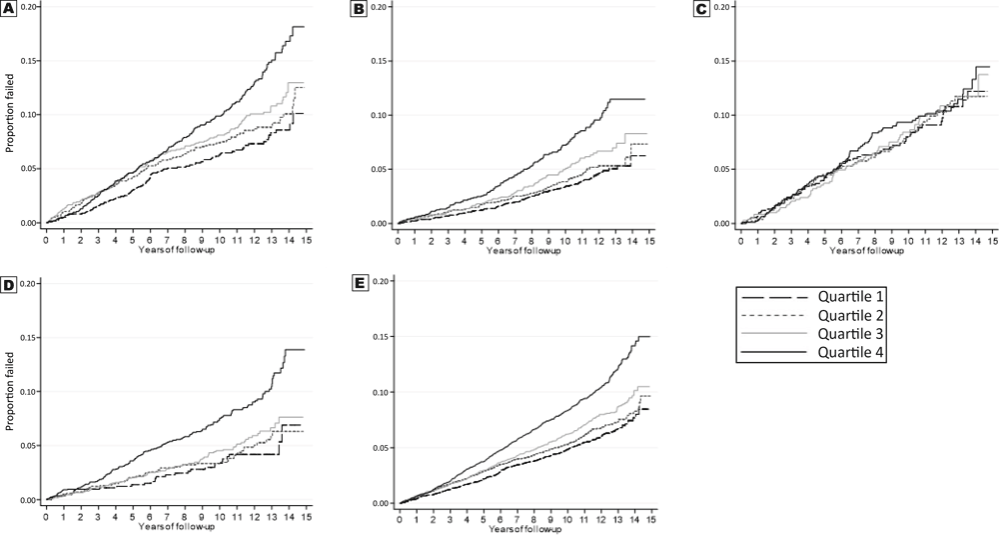
Kaplan–Meier incident coronary heart disease event estimates, by quartile of waist circumference among participants in the REasons for Geographic and Racial Differences in Stroke Study. A, White men. B, White women. C, African American men. D, African American women. E, Total sample.

Kaplan–Meier curves for the overall sample and race–sex subgroups show that, except for African American men, quartile 4 for waist circumference had an increased risk of incident CHD (Figure 2).

## Discussion

In this study using REGARDS study cohort data, we assessed the influence of both individual and neighborhood characteristics on the relationship between waist circumference and incidence of CHD in the overall cohort and among race–sex subgroups. Although the relationship between incident CHD and sociodemographic factors is well documented, we did not observe that individual or neighborhood sociodemographic or health services characteristics influenced the relationship between waist circumference and incident CHD in the overall sample or in the race–sex stratified models. Among White men and women and African American women, having a waist circumference in the highest quartile was associated with increased risk of incident CHD. We found variation in the association between the highest-quartile waist circumference group and incident CHD across race–sex subgroups. The adjusted HR of incident CHD among African American women in the highest quartile of waist circumference was higher than that among White men in the highest quartile of waist circumference (adjusted HR, 1.88 vs 1.56). We found no association between waist circumference and incident CHD among African American men in the sample.

The finding of variation of the risk of high waist circumference on incident CHD across race–sex subgroups observed here was similar to findings in a previous study that used structural equation modeling to construct metabolic risk profiles of individual metabolic risk factors for CVD mortality in race–sex subgroups. Mercado et al found waist circumference among White men and women was less associated with CVD mortality than among African American women, although the HR among African American women was not significant (9). Our results reaffirmed well-established disparities in waist circumference and CHD among race–sex subgroups.

Curiously, we found no association between waist circumference and incident CHD among African American men. This result is concordant with findings from the structural equation modeling work of Mercado et al, which also did not note an association between waist circumference and CVD mortality among African American men (9). One possible explanation for this lack of association may be that elevated blood pressure, which is highly prevalent among African American men (39), may overwhelm waist circumference as a risk factor for incident CHD. Although we adjusted for hypertension treatment in our analysis, we did not adjust for measured blood pressure levels. Compared with the general US population with hypertension, African American men are less likely to be treated to achieve goal blood pressure (40) and they have lower rates of adherence to treatment regimens for hypertension (41). Additionally, in our study, African American men were significantly more likely than other subgroups to lack a usual source of health care, which may have given them fewer opportunities to manage risk factors for CHD. Usual source of care has been associated with reduced overall mortality and better health outcomes (42), including cardiovascular health outcomes (43). African American men in our study had significantly lower rates of health insurance coverage compared with the overall sample (90.0% vs 95.7%, *P* < .001), which may have further limited exposure to cardiovascular risk factor reduction via preventive care, health behavior interventions, and medication adherence. Another REGARDS study noted racial differences in fatal and nonfatal CHD among race–sex subgroups: African American men had a higher risk of fatal CHD but a lower risk of nonfatal CHD compared with White men, and African American women had a lower risk of fatal CHD but a higher risk of nonfatal CHD compared with White women (34). These findings point to potential underlying differences that may contribute to the findings here. The ways in which smoking, depression, untreated hypertension, joblessness, and poor living conditions may be more strongly associated than waist circumference with CHD among African American men should be further explored.

Another potential explanation for the lack of association we observed among African American men between increased waist circumference and incident CHD would be that stress caused by structural racism may be unmeasured in our model and driving incident CHD in this subpopulation (44). Although we did include a measure of neighborhood African American–White segregation in our analysis, it did not impact the relationship between waist circumference and incident CHD in the overall sample or in race–sex subgroups. Other spatial measures of structural racism have been developed that may be better suited to measure the impact of place-based structural racism on health outcomes (45). Although no association was found in this study, others have noted a weathering effect of sustained levels of social and environmental stressors caused by structural racism that negatively impact the health of African Americans, particularly African American men (46).

Our analysis did not find any significant interaction effects between neighborhood environment and waist circumference on CHD in the full sample, but poverty was identified as a potentially important factor that influenced the relationship between waist circumference and CHD among African American and White women in this study, with high–poverty neighborhood environments carrying a higher risk of CHD among women in the highest quartile of waist circumference and CHD. Further work should explore how poverty influences CHD risk among women in the context of central adiposity.

Our study has several strengths, including the large national scope of the REGARDS study, which may have enhanced generalizability, and rigorously adjudicated study end points. It also has some limitations. Waist circumference was the main exposure variable in our study and was measured only at the beginning of the study. However, change in waist circumference over time has not been shown to be a better predictor of incident CHD than baseline waist circumference (47,48). Although REGARDS participants represent a national sample of African American and White adults, our results may not be generalizable to all populations because some racial and ethnic groups were not represented in the study. Lastly, it is important to note that many of the baseline demographic covariates in this analysis were self-reported, and such responses are subject to response and recall bias.

Our study found that higher waist circumference was associated with higher risk of incident CHD for all race–sex groups except African American men. We did not observe any neighborhood or individual characteristics that attenuated the association between waist circumference and incident CHD in the fully adjusted overall sample or in fully adjusted race–sex stratified models. Further work is needed to explore the unexpected lack of association between waist circumference and incident CHD among African American men.

## Appendix List of Covariates and Literature Supporting the Established Association Between Covariates and Coronary Heart Disease and Obesity

**Table:**

Characteristic	Reference
**Individual**	
Age	North BJ, Sinclair DA. The intersection between aging and cardiovascular disease. Circ Res 2012;110(8):1097–108.
Gender	Rodgers JL, Jones J, Bolleddu SI, Vanthenapalli S, Rodgers LE, Shah K, et al. Cardiovascular risks associated with gender and aging. J Cardiovasc Dev Dis 2019;6(2):19.
Race	Bell CN, Thorpe RJ Jr, Bowie JV, LaVeist TA. Race disparities in cardiovascular disease risk factors within socioeconomic status strata. Ann Epidemiol 2018;28(3):147–52.
Marital status	Wong CW, Kwok CS, Narain A, Gulati M, Mihalidou AS, Wu P, et al. Marital status and risk of cardiovascular diseases: a systematic review and meta-analysis. Heart 2018;104(23):1937–48.
Region of residence	Tabb LP, Ortiz A, Judd S, Cushman M, McClure LA. Exploring the spatial patterning in racial differences in cardiovascular health between Blacks and Whites across the United States: the REGARDS study. J Am Heart Assoc 2020;9(9):e016556.
Educational attainment	Hamad R, Nguyen TT, Bhattacharya J, Glymour MM, Rehkopf DH. Educational attainment and cardiovascular disease in the United States: a quasi-experimental instrumental variables analysis. PLoS Med 2019;16(6):e1002834.
Income	Odutayo A, Gill P, Shepherd S, Akingbade A, Hopewell S, Tennankore K, et al. Income disparities in absolute cardiovascular risk and cardiovascular risk factors in the United States, 1999–2014. JAMA Cardiol 2017;2(7):782–90.
Health insurance status	Pancholy S, Patel G, Pancholy M, Nanavaty S, Coppola J, Kwan T, et al. Association between health insurance status and in-hospital outcomes after ST-segment elevation myocardial infarction. Am J Cardiol 2017;120(7):1049–54.
Usual source of care	Spatz ES, Ross JS, Desai MM, Canavan ME, Krumholz HM. Beyond insurance coverage: usual source of care in the treatment of hypertension and hypercholesterolemia. Data from the 2003–2006 National Health and Nutrition Examination Survey. Am Heart J 2010;160(1):115–21.
Smoking status	Tonstad S, Johnston JA. Cardiovascular risks associated with smoking: a review for clinicians. Eur J Cardiovasc Prev Rehabil 2006;13(4):507–14.
Diabetes diagnosis	Resnick HE, Howard BV. Diabetes and cardiovascular disease. Annu Rev Med 2002;53(1):245–67.
Hypertension diagnosis	Sowers JR, Epstein M, Frohlich ED. Diabetes, hypertension, and cardiovascular disease: an update. Hypertension 2001;37(4):1053–9.
Lipid disorder diagnosis	Michos ED, McEvoy JW, Blumenthal RS. Lipid management for the prevention of atherosclerotic cardiovascular disease. N Engl J Med 2019;381(16):1557–67.
**Ecologic**	
Primary care physician supply	Gaglioti AH, Petterson S, Bazemore A, Phillips R. Access to primary care in US counties is associated with lower obesity rates. J Am Board Fam Med 2016;29(2):182–90.
Neighborhood poverty	Topel ML, Kim JH, Mujahid MS, Sullivan SM, Ko YA, Vaccarino V, et al. Neighborhood socioeconomic status and adverse outcomes in patients with cardiovascular disease. Am J Cardiol 2019;123(2):284–90.
Area segregation	Kershaw KN, Albrecht SS. Racial/ethnic residential segregation and cardiovascular disease risk. Curr Cardiovasc Risk Rep 2015;9(3):10.
